# Correlation between morphological parameters and dosimetric parameters of the heart and spinal cord in the intermediate‐ and advanced‐stage esophageal cancer

**DOI:** 10.1002/cnr2.2015

**Published:** 2024-03-15

**Authors:** Wenjuan Zhao, Linzhen Lan, Bichun Xu, Di Chen, Yusha Zeng, Feibao Guo, Huojun Zhang

**Affiliations:** ^1^ School of Medical Instrument and Food Engineering University of Shanghai for Science and Technology Shanghai China; ^2^ Department of Radiation Therapy, Cancer Center The First Affiliated Hospital of Fujian Medical University Fuzhou China; ^3^ Graduate School The Navy Medical University Shanghai China; ^4^ Fujian Provincial Hospital Fuzhou China; ^5^ Key Laboratory of Radiation Biology of Fujian higher education institutions, the First Affiliated Hospital Fujian Medical University Fuzhou China

**Keywords:** correlation, dosimetric parameters, esophageal cancer, IMRT, OAR

## Abstract

**Background:**

Radiation therapy plays a pivotal role as the primary adjuvant treatment for esophageal cancer (EPC), emphasizing the critical importance of carefully balancing radiation doses to the target area and organs at risk in the radiotherapeutic management of esophageal cancer.

**Aims:**

This study aimed to explore the correlation between morphological parameters and dosimetric parameters of the heart and spinal cord in intermediate‐ and advanced‐stage esophagus cancer to provide a reference for clinical treatment.

**Methods and results:**

A total of 105 patients with intermediate‐ and advanced‐stage EPC, who received treatment in our hospital from 2019 to 2021, were included. The morphological parameters were calculated by imaging. Intensity‐modulated radiation therapy plan was executed at Raystation4.7. The PTV‐G stood for the externally expanded planning target volume (PTV) of the gross tumor volume (GTV) and PTV‐C for the externally expanded volume of the clinical target volume (CTV). The prescription dose of PTV‐G and PTV‐C was set as 60Gy/30F and 54Gy/30F, respectively. The linear regression model was used to analyze the correlation between morphologic parameters of EPC and dosimetric parameters of the heart and spinal cord. In 105 cases, the total lung length was correlated with the spinal cord maximum dose (D_2_). The heart mean doses (D_mean_) and heart V_40_ (the relative volume that receives 40 Gy or more) was correlated with PTV‐G volume, PTV‐G length; In middle‐ and upper‐segment EPC cases, only the total lung volume was correlated with the spinal cord D_mean_, spinal cord D_2_, heart D_mean_, and heart V_40_; In middle‐stage EPC cases, the heart D_mean_ was correlated with the PTV‐G volume, PTV‐G length. The total lung length was correlated with the spinal cord D_2_; In middle‐ and lower‐segment EPC, only the PTV‐G volume and PTV‐G length were correlated with the heart D_mean_. All the aforementioned values were statistically significant.

**Conclusions:**

Combined with the unsegmented tumor and different locations, the organ at risk dose was comprehensively considered.

## 1 INTRODUCTION

Esophageal cancer (EPC) is one of the most common clinical gastrointestinal malignancies and the eighth most common malignant tumor globally. It usually occurs in the esophageal epithelium; 70%–80% of EPC occurs in Asia, especially in China. Esophageal squamous cell carcinoma accounts for about 90% of EPC cases. Most patients with EPC are in the advanced stage by the time of treatment. Only chemotherapy, radiotherapy, and molecular targeted therapy can be used for advanced‐stage EPC, but the treatment effect is not good.^1^, ^2^, ^3^, ^4^ Radiotherapy has been widely used in esophageal cancer radiotherapy. It is the main treatment for patients with intermediate‐ and advanced‐stage EPC. Radiation‐induced heart damage (RIHD) is one of the common complications of radiotherapy for middle and lower thoracic esophageal cancer.^5^, ^6^ Intensity‐modulated radiation therapy (IMRT) can concentrate radiation in specific areas of the tumor with imaging technology, thus improving the radiation dose of the tumor and effectively reducing the radiation damage of the surrounding normal tissues.^7^ IMRT can effectively reduce the dose of exposure to organs at risk, such as double lungs, heart, and spinal cord, on the basis of highly conforming target area of radiotherapy, resulting in less radiation damage; hence, it has been widely used.^8^ Therefore, radiation therapy should not only ensure the amount of irradiation in the target area but also control the damage caused by radiation to the heart to reduce the risk of RIHD.^9^ Heart exposure volume and radiation dose are the most important influencing factors in radiation heart injury. In addition, Myelosuppression may occur in patients with esophageal cancer receiving radical radiotherapy, especially concurrent radiotherapy and chemotherapy.^10^ The spinal cord is a critical organ to keep under tolerance dose. Radiation‐induced spinal cord injury from excessive irradiation will have serious consequences. The dose limit is 50 Gy to spinal cord from guidelines,^11^ but in clinical practice the limit is usually set to be 45 Gy due to the seriousness of the side effect. Although modern radiation therapy techniques have been introduced to clinical practice, the dose distribution in many cases of esophageal cancer continues to be a compromise between sparing of the lungs, heart and spinal cord.^12^ Therefore, the heart and spinal cord are usually considered the significant organ at risk for the radiation of esophageal cancer.

In UICC/AJCC staging system,^13^ tumor location was first brought into 7th edition staging system for esophageal cancer, which indicated that tumor location could affect the prognosis. Studies^14^, ^15^ from Japan have shown tumors in the upper portion of the esophagus to be independently associated with recurrence and mortality. According to primary tumor location, the highest risk of heart disease related death was for mid‐esophageal tumors.^16^ Gharzai et al.^17^ found that there was higher probability of cardiac death among patients after radiotherapy compared to no radiation group and in lower esophageal carcinomas compared to upper and middle esophageal carcinomas. Spinal cord irradiation can result in acute transient myelopathy due to demyelination manifesting as Lhermitte syndrome. Late effects include lower motor neuron syndrome, telangiectasias, and subsequent hemorrhage.^18^

Through correlating dosimetry and morphology in intermediate‐ and advanced‐stage esophageal cancer, this study offers clinical guidance and aims to minimize radiation toxicity in the treatment of esophageal cancer. We have previously shown that the ACSA (axial cross‐sectional area) of tumors (PTV volume/PTV length) had a greater influence on the lung high‐dose area than the lung low‐dose area.^19^ The present study examines the detailed correlation between morphological parameters and dosimetry parameters of the heart and spinal cord in patients with middle to advanced esophageal cancer.

## 2 MATERIALS AND METHODS

### 2.1 Inclusion and exclusion criteria

Inclusion criteria: (1) patients diagnosed according to the AJCC eighth edition cancer staging system IIIA‐IVB, (2) first visit and treatment in our hospital, (3) complete clinical data, and (4) completed chemotherapy cycle. Exclusion criteria: (1) infectious and congenital diseases, (2) death during treatment, and (3) no serious heart, brain, liver, or kidney diseases.

### 2.2 Method of measurement

#### 2.2.1 Total lung volume measurement

**FIGURE 1:**
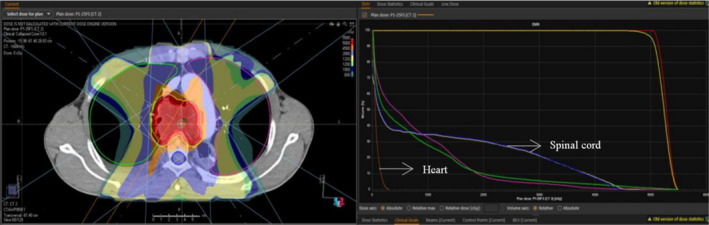
Esophageal cancer DVH plot based on Raystation system.

The whole lung was sketched with the automatic sketching tool that comes with the Treatment Planning System (TPS) software for Raystation 4.7 (Figure 1), and then manually modified to exclude the trachea and bronchi. Lung tissue is a “parallel organ” consisting of many “functional units” that have the same function and are relatively independent of each other. The dose of lung radiation tolerance was determined by the extent of exposure. The larger the exposure range of the lung, the smaller the tolerance of the lung, that is, the larger the lung volume effect.

#### 2.2.2 Simulated positioning

All patients were fixed in supine position with Claridis thermoplastic, with a C‐shaped pillow supporting the head, legs together, and arms at the sides of the body. and a spiral computed tomography (CT) scan was performed with the Siemens large‐aperture CT simulation positioning machine SOMATOM Definition AS, ranging from the first cervical vertebra to the level of the lower border of the liver. The scanning slice thickness was 3–5 mm. The images were positioned and imported into the Radiation Station TPS.

#### 2.2.3 Measurement of the tumor target volume

Tumor target volume (GTV): The GTV of EPC was well defined. The length of the primary tumor was mainly based on esophagography, esophagoscopy, intracavitary ultrasound, and other endoscopic results, and was delineated by CT images. GTV should include esophageal wall thickness greater than 0.5 cm or non‐air cavity diameter greater than 1.0 cm. Since supraclavicular, mediastinal lymph node metastases, and distant metastases were excluded, the target profiles of lymph nodes and metastases were not involved.

### 2.3 Processing method

A total of 105 patients with EPC were given the same prescription dose (the PTV‐G prescription dose was set at 60 Gy/30 F, and the PTV‐C prescription dose was set at 54 Gy/30 F), and IMRT was designed under the same conditions to strictly control the quality of radiotherapy plan. The dose limits of peripheral organs at risk were determined according to the ICRU83 report and optimized using the iterative convolution algorithm.

### 2.4 Parameter record

The morphological parameters, such as PTV‐G length, PTV‐G volume, total lung volume, and total lung length, were measured on CT images by clinicians. The spinal cord D_2_, spinal cord D_mean_, heart D_mean_, heart V_40_, and other dosimetric parameters were calculated according to the dose‐volume histograms.

### 2.5 Statistical analysis

SPSS19 was used for statistical analyses. The morphological parameters, such as PTV‐G length, PTV‐G volume, lung volume, and lung length, and the dosimetric parameters, such as spinal cord D_2_, spinal cord D_mean_, heart D_mean_, and heart V_40_, were analyzed using the linear regression model. The method of fitting the regression equation was least square estimation to judge whether a linear relationship existed between independent and dependent variables. Therefore, we established the hypothesis H0: *β* = 0 and H1: *β* = 0, using the *t* test, when *p* ≤ .05 indicated a statistically significant difference.

## 3 RESULTS

### 3.1 Patients' characteristics

A total of 105 patients with advanced‐ and intermediate‐stage EPC(We define intermediate‐ and advanced‐stage esophageal cancer as pathological stage III and IV, excluding early stages including pathological stage I and II.^20^, ^21^), aged 69–93 years, were included, of whom 80 (76.190%) were male and 25 (23.810%) were female (Table 1). In this study, upper esophagus was defined with primary site codes C15.0 (cervical esophagus) and C15.3 (upper third of esophagus). Code C15.4 (middle third of esophagus) was used to identify the middle esophagus. Lower esophagus was a combination of codes C15.2 (abdominal esophagus) and C15.5 (lower third of esophagus).^22^, ^23^, ^24^

**TABLE 1:** Clinical data of the patients.

Clinical index	Parameter
Age (year)	66.415 ± 13.541
Sex	
Male (cases)	80
Female (cases)	25
Tumor location	
Upper segment (cases)	23
Middle section (cases)	46
Lower segment (number of cases)	36
Total lung volume (cm^3^)	3159.230 ± 887.343
Total length of the lung (cm)	18.046 ± 4.288
PTV‐G length (cm)	6.532 ± 3.242
PTV‐G volume (cm^3^)	96.041 ± 73.385
Volume of the heart (cm^3^)	563.076 ± 136.146

**FIGURE 2:**
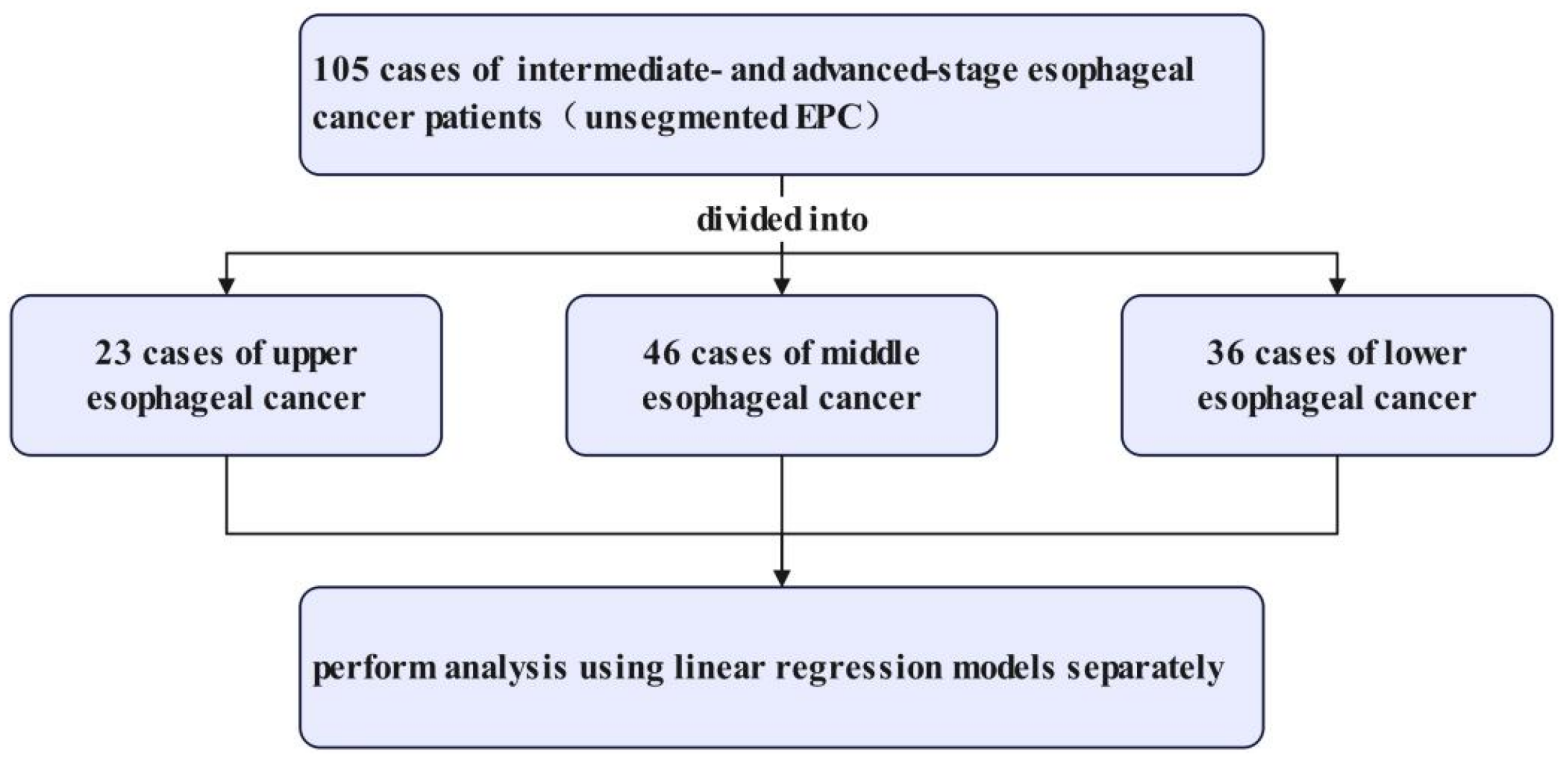
The flow chart of our study.

A total of 105 esophageal cancer patients (unsegmented EPC) were analyzed using the linear regression model. Subsequently, the 105 patients were divided into upper, middle, and lower segments and individually analyzed using the linear regression model (Figure 2). The statistical analysis results were comprehensively discussed in relation to the correlation between dose‐volume parameters of the heart and spinal cord in patients with advanced esophageal cancer at different locations, and their morphological relationships with the heart, lungs, and spinal cord.

### 3.2 Linear regression model analysis results

**FIGURE 3:**
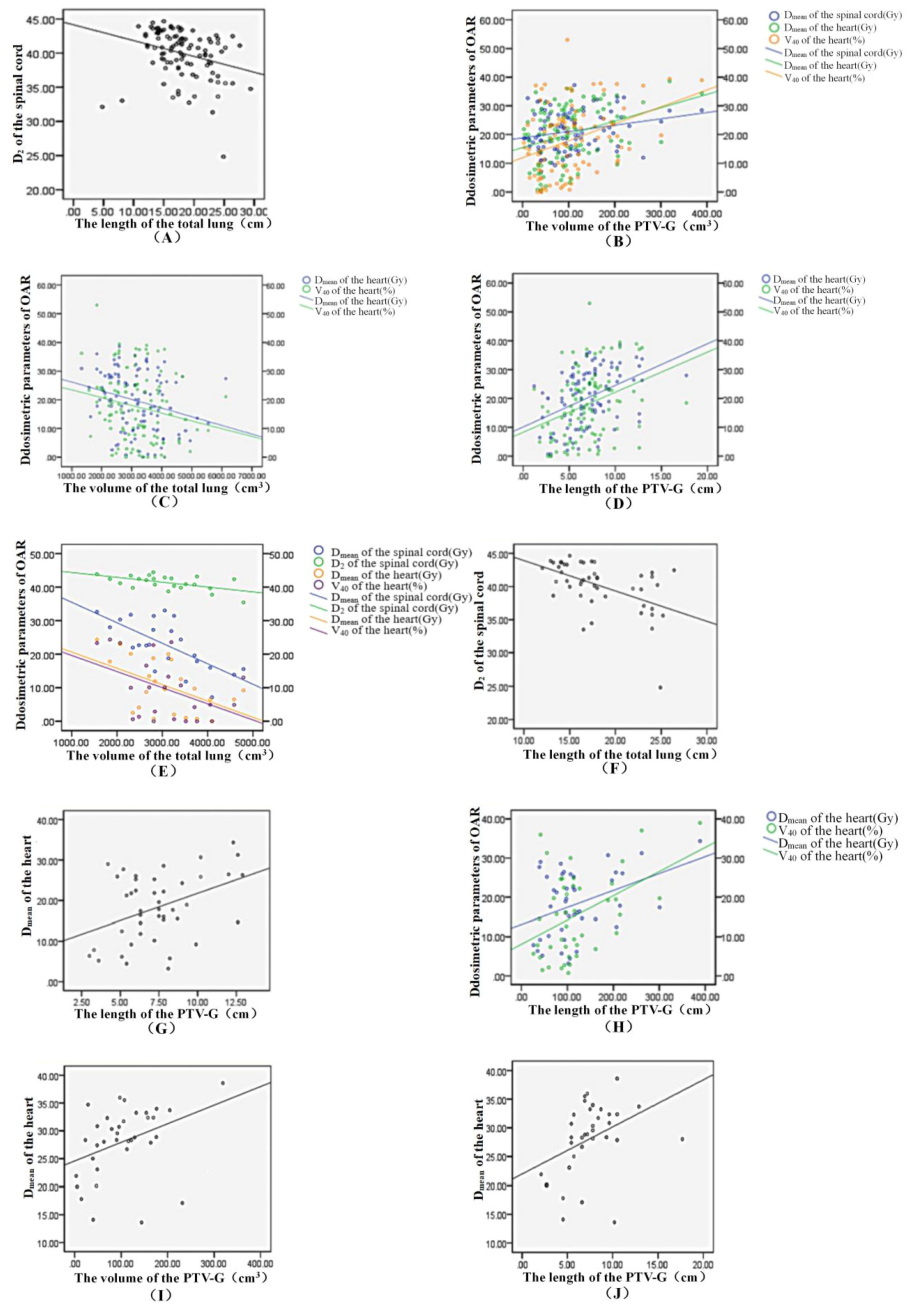
Scatter plot of the total lung length and spinal cord D_2_ in unsegmented EPC (A); Scatter plot of the PTV‐G volume and spinal cord D_mean_, heart D_mean_, heart V40 in unsegmented EPC (B); Scatter plot of the total lung volume and heart D_mean_, heart V_40_ in unsegmented EPC (C); Scatter plot of the PTV‐G length and heart D_mean_, heart V_40_ in unsegmented EPC (D); Scatter plots of the total lung volume and spinal cord D_mean_, spinal cord D2, heart D_mean_, and heart V_40_ in upper EPC (E); Scatter plots of the total lung length and spinal cord D_2_ in middle EPC (F); Scatter plots of the PTV‐G length and heart D_mean_ in middle EPC (G); Scatter plots of the PTV‐G volume and heart D_mean_, heart V_40_ in middle EPC (H); Scatter plots of the PTV‐G volume and heart D_mean_ in lower EPC (I); Scatter plots of the PTV‐G length and heart D_mean_ in lower EPC (J).

For 105 cases of unsegmented esophageal cancer patients: The results of linear regression showed that the total lung length was negatively correlated with the spinal cord D_2_ (*p* = .003, *r* = .285) (Figure 3A). The total lung volume was negatively correlated with the heart D_mean_ (*p* = .007, *r* = .263) and heart V_40_ (*p* = .042, *r* = .200) (Figure 3C). The PTV‐G volume was positively correlated with the spinal cord D_mean_ (*p* = .019, *r* = .237), heart D_mean_ (*p* = .001, *r* = .335), and heart V_40_ (*p* = .000, *r* = .356) (Figure 3B). PTV‐G length was positively correlated with the heart D_mean_ (*p* = .000, *r* = .420) and heart V_40_ (*p* = .001, *r* = .338) (Figure 3D). A strong correlation was found between heart D_mean_ and lung volume, PTV‐G volume, and PTV‐G length (*p* < .01). A strong correlation was observed between spinal cord D_2_ and lung length (*p* < .01). A strong correlation was found between heart V_40_ and PTV‐G length (*p* < .01) (Table 2).

**TABLE 2:** Analysis of linear regression results of 105 cases with unsegmented EPC.

Predicted variable	Variable in the regression equation	Correlation coefficient (r)	*p*
Spinal cord D_2_	Total length of the lung	.285	.003
Heart D_mean_	Total volume of the lung	.263	.007
Heart V_40_	Total volume of the lung	.200	.042
Spinal cord D_mean_	PTV‐G volume	.237	.019
Heart D_mean_	PTV‐G volume	.335	.001
Heart V_40_	PTV‐G volume	.356	.000
Heart D_mean_	PTV‐G length	.420	.000
Heart V_40_	PTV‐G length	.338	.001

For 23 cases of upper esophageal cancer patients: Only the total lung volume was negatively correlated with the spinal cord D_mean_ (*p* = .000, *r* = .686), spinal cord D_2_ (*p* = .005, *r* = .561), heart D_mean_ (*p* = .011, *r* = .521), and heart V_40_ (*p* = .032, *r* = .032) (Figure 3E). The total lung volume was strongly correlated with the spinal cord D_mean_ and spinal cord D_2_ (*p* < .01) (Table 3).

**TABLE 3:** Analysis of linear regression results of 23 cases of upper EPC.

Predicted variable	Variable in the regression equation	Correlation coefficient (r)	*p*
Spinal cord D_2_	Total volume of the lung	.561	.005
Spinal D_mean_	Total volume of the lung	.686	.000
Heart D_mean_	Total volume of the lung	.521	.011
Heart V_40_	Total volume of the lung	.449	.032

For 46 cases of middle esophageal cancer patients: The total lung length was negatively correlated with the spinal cord D_2_ (*p* = .000, *R* = 0.506) (Figure 3F), the PTV‐G length was positively correlated with the heart D_mean_ (*p* = .007, *r* = .409) (Figure 3G), the PTV‐G volume was negatively correlated with the heart D_mean_ (*p* = .010, *r* = .389), and the heart V_40_ was positively correlated (*p* = .003, *r* = .440) (Figure 3H). The total lung length was strongly correlated with the spinal cord D_2_ (*p* < .01), the PTV‐G length was strongly correlated with the heart D_mean_ (*p* < .01), and the PTV‐G volume was strongly correlated with the heart V_40_ (*p* < .01) (Table 4).

**TABLE 4:** Analysis of linear regression results of 46 cases of middle EPC.

Predicted variable	Variable in the regression equation	Correlation coefficient (r)	*p*
Spinal cord D_2_	Total length of the lung	.506	.000
Heart D_mean_	PTV‐G length	.409	.007
Heart D_mean_	PTV‐G volume	.389	.010
Heart V_40_	PTV‐G volume	.440	.003

For 36 cases of lower esophageal cancer patients: only the PTV‐G volume (*p* = .027, *r* = .374) and PTV‐G length (*p* = .019, *r* = .394) were positively correlated with the heart D_mean_ (Figure 3I,J; Table 5).

**TABLE 5:** Analysis of linear regression results of 36 cases of lower EPC.

Predicted variable	Variable in the regression equation	Correlation coefficient (r)	*p*
Heart D_mean_	PTV‐G volume	.374	.027
Heart D_mean_	PTV‐G length	.394	.019

## 4 DISCUSSION

The effect of radiotherapy on esophageal cancer is still unsatisfactory; the literature reports that the 5‐year survival rate of radiotherapy is only 10%.^25^ The substantial improvement in survival after radiotherapy in patients with esophageal cancer may be related to the lower radiation exposure of the heart.^26^ Radiation heart injury is currently one of the most serious adverse reactions of radiotherapy for thoracic esophageal cancer; it is also the main reason for the low survival rate of patients.^27^ Groves et al.^9^ found that V_30_ and V_40_, the percentages of the heart receiving a high dose of radiation in the total volume of the heart, were important predictors of induced heart radiation injury. Retrospective studies at other single institutions also confirmed the association of high heart dose with reduced overall survival. Between 40 and 50 Gy, the dose–response curve for the cardiotoxicity of esophageal cancer showed a steep part. A vast majority of studies on esophageal cancer have been reported regarding pericardial diseases and various dose parameters. Although constrictive pericarditis has been reported in both early and late stages, the prognosis of esophageal cancer is generally poor. Also, the development of ischemic heart disease over several years and decades is not considered.^28^

The incidence of radiation heart injury is closely related to the radiation volume of the heart. The incidence of radiation‐induced heart injury is high when the radiation volume is large, and low when the radiation volume is small. Radiation‐induced heart injury is a non‐negligible adverse reaction in patients with esophageal cancer; studies have shown myocardial injury in the early stage of esophageal cancer radiotherapy.^29^, ^30^ The ratio of irradiated to the total heart volume and the absolute heart's exposure to radiation was significantly higher in patients who developed a cardiac event. The authors found volume cut‐off of 280 mL of the heart irradiated with a dose higher than 50 Gy (V_50_) with a risk ratio of 16.80.^31^ Ogino et al.^32^ found that the pericardial V50 in patients with symptomatic effusion ranged between 17.1% and 21.7%; therefore, the authors recommend the threshold for the pericardial V50 ≤ 17%. Wei et al.^33^ found that the risk of pericardial effusion increased significantly with a mean pericardial dose of 26.1 Gy and V30 of the pericardium greater than 46%.

Tumor length was previously described as a prognostic factor for survival^.^ ^34^, ^35^ Ai et al.^22^ found that the different impact of tumor location of esophageal cancer on liver or lung metastasis. Upper esophageal cancer was more relevant to lung metastasis while lower esophageal cancer to liver metastasis. Both tumor and nodal stage and lesion length had a significant influence on OS, with patients having T1/2 tumors or N0 status achieving a median OS of 25.9 and 29.6 months, respectively.^36^ Zhu et al.^37^ found that in univariate analysis, tumor length ≤4 cm favored recurrence‐free survival. Patients with tumor location at middle 1/3 or length ≤5 cm tended to gain survival benefit from RT, while those with tumor location at lower 1/3 or length >5 cm were priorly recommended to receive surgical resection as local therapy.^38^

In this study, the relationship between morphology and dosimetry of the heart and spinal cord was studied in the case of mid‐advanced EPC cases with or without segmentation. The following conclusions were drawn: (1) Spinal cord D_2_: only the total lung volume in the case of unsegmented EPC, the total lung volume in the case of the middle‐upper segment, and the total lung length in the case of the middle segment should be considered. (2) Spinal cord D_mean_: Only the PTV‐G volume without tumor segmentation and the total lung volume in the middle and upper segments should be taken into account. (3) Heart D_mean_: The PTV‐G volume and PTV‐G length when the tumor is unsegmented and located in the middle, as well as the total lung volume when the tumor is located in the middle and upper segments and the PTV‐G volume when the tumor is located in the middle and lower segments, should be considered. (4) Heart V_40_: Only the PTV‐G volume and PTV‐G length without tumor segmentation, the total lung volume in the upper‐middle segment, and the PTV‐G volume in the middle segment should be considered.

This study analyzed the findings on radiotherapy for the heart and spinal cord in advanced EPC cases. When the tumor was unsegmented, the heart dose was mainly related to the total lung volume and the volume and shape of the PTV‐G, while the spinal cord dose was related to the total lung length and PTV‐G volume. This might be because the anatomical position of the esophagus is close to the heart and spinal cord. The radiation received by the heart and spinal cord increased with the increase in tumor volume and length. The lungs are located on both sides of the esophagus and are closely related to the heart and spinal cord. This also had a certain influence on the dose of the heart and spinal cord. When the tumor was located in the middle and upper segments, the dose of the heart and spinal cord was only related to the total lung volume. The volume directly adjacent to the lung and the esophagus was larger, where the heart was not completely in the same plane as the tumor. Therefore, the volume of the heart and spinal cord was affected by the volume of the lung. When the tumor was located in the middle and lower segments, the heart dose was only related to the tumor volume and length. This may be because the middle and lower segments of the esophagus were located at the junction of the tracheal bifurcation plane and the esophagus and stomach, and most of the heart was in between. Over time, the radiation to the heart also increased. The middle esophagus and lower esophagus were close to the heart and lungs, and radiotherapy inevitably resulted in higher radiation doses to the heart and lungs.^39^ In addition, compared with unsegmented EPC cases, when the tumor was located in the mid‐esophagus, the effect of total lung length on the spinal cord D_2_ was greater. The spinal cord D_2_ of EPC cases located in the middle esophagus decreased by 42.995 Gy and 46.207 Gy, respectively. Compared with the tumor located in the middle segment, the PTV‐G volume and PTV‐G length had a greater impact on the heart D_mean_ when the tumor was located in the middle and lower segments. The scatter plot linear regression equation showed that when the length of PTV‐G increased by 5 cm, the D_mean_ of the tumor located in the middle and lower segments of the heart increased by 15.065Gy and 26.046 Gy, respectively. When the volume of PTV‐G increased by 100 cm^3^, the D_mean_ of the tumor located in the middle and lower segments of the heart increased by 17.402 Gy and 17.402 Gy, respectively.

When radiotherapy was performed for patients with intermediate‐ and advanced‐stage EPC, the relationship between total lung volume, lung length, PTV‐G volume, PTV‐G length, spinal cord D_mean_, spinal cord D_2_, heart D_mean_, and heart V_40_ should be considered comprehensively first, and then the relationship between data of each group should be considered in segments. The findings of this study might provide guidance for clinical treatment and physical planning.

The objective of this study is to provide therapists with clinical guidance and references by analyzing various aspects of esophageal cancer statistics. This will enable them to improve prediction accuracy and establish precise definitions of dosimetric limits for organs at risk in radiotherapy. In contrast to previous research, which primarily focused on analyzing the correlation between unsegmented esophageal cancer (PTV‐G volume and PTV‐G length) and pulmonary dose‐volume parameters, as well as the ACSA (axial cross‐sectional area) of tumors (PTV volume/PTV length) and pulmonary dose‐volume parameters, this study takes a different approach. The current study selected the spinal cord and heart as the subjects of analysis, conducting a systematic and comprehensive examination of the effects of radiation dose on these organs within the context of esophageal cancer. This analysis included the association of the heart and spinal cord with PTV‐G length, PTV‐G volume, total lung volume, and total lung length in four conditions (unsegmented, segmented upper, middle, and lower end), thereby addressing numerous deficiencies in previous literature regarding the factors that impact the heart and spinal cord. The limitation of this study lies in the small number of cases, which necessitates an increase in sample size for subsequent work to enhance prediction accuracy.

## AUTHOR CONTRIBUTIONS

**Wenjuan Zhao:** Data curation (equal); methodology (equal); writing – original draft (equal); writing – review and editing (equal). **Linzhen Lan:** Data curation (equal); methodology (equal). **Bichun Xu:** Data curation (equal); validation (equal). **Di Chen:** Data curation (equal); investigation (equal); methodology (equal). **Yusha Zeng:** Data curation (equal). **Feibao Guo:** Data curation (equal); methodology (equal); writing – original draft (equal); writing – review and editing (equal). **Huojun Zhang:** Investigation (equal); methodology (equal); supervision (equal); writing – review and editing (equal).

## CONFLICT OF INTEREST STATEMENT

The authors have stated explicitly that there are no conflicts of interest in connection with this article.

## ETHICS STATEMENT

This work has been carried out in accordance with the Declaration of Helsinki (2000) of the World Medical Association. This study was approved by Department of Radiation Therapy, Cancer Center, The First Affiliated Hospital of Fujian Medical University. This article is a retrospective study. Therefore the Institutional waived the requirement to obtain distinct written informed consent from the patients.

## UNBLINDED STATEMENT

Our trial is a open trial, a total of 105 patients with middle‐and advanced‐stage EPC, who received treatment in our hospital from 2019 to 2021, were included. Intensity‐modulated radiation therapy plan was executed at Raystation4.7. The prescription dose of PTV‐G and PTV‐C was set as 60 Gy/30 F and 54 Gy/30 F, respectively. We collectet the parameter record such as the morphological parameters, such as PTV‐G length, PTV‐G volume, total lung volume, and total lung length, were measured on CT images by clinicians. The spinal cord D2, spinal cord Dmean, heart Dmean, heart V40, and other dosimetric parameters were calculated according to the dose volume histograms. The linear regression model was used to analyze the correlation between morphologic parameters of EPC and dosimetric parameters of the heart and spinal cord.

## Data Availability

The data that support the findings of this study are available from the corresponding author upon reasonable request.
